# A Descriptive Study on the Role of Magnetic Resonance Imaging in Staging Avascular Necrosis of the Hip Joint: Current Trends and Insights

**DOI:** 10.7759/cureus.86867

**Published:** 2025-06-27

**Authors:** Bhawna Rohilla, Lavanya Dharmalingam, G. Kamalakannan, Prabhu C S.

**Affiliations:** 1 Radiology, Aarupadai Veedu Medical College, Puducherry, IND; 2 Orthopaedics, Aarupadai Veedu Medical College, Puducherry, IND

**Keywords:** arco classification, avascular necrosis of the hip, covid-19 and avascular necrosis hip, ficat and arlet staging, hip mri, musculoskeletal mri, osteonecrosis

## Abstract

Background: Avascular necrosis (AVN) of the hip, also known as osteonecrosis or ischemic necrosis, is cellular necrosis of bone and marrow elements. Although clinical evaluation is the first step in assessing the hip joint, imaging is fundamental for accurate diagnosis and staging of AVN.

Objective: We aim to assess the role of magnetic resonance imaging (MRI) in evaluating AVN of the hip, describe imaging features, and classify AVN using various classification systems.

Methods: A cross-sectional study was conducted over two years at the Department of Radiodiagnosis, Aarupadai Veedu Medical College and Hospital, Puducherry, a tertiary healthcare center situated in Southern India. The study included 50 patients referred for MRI of the hip joint with suspected unilateral or bilateral hip joint pathology, with or without pain. The participants' demographic data, clinical presentations, and MRI findings were recorded. Data were collected, and Statistical Package for the Social Sciences version 28 (IBM Corp., Armonk, NY) was used for all statistical analyses.

Results: The study comprised 50 patients, with a significant male predominance (62%, n=31) and a male-to-female ratio of 1.63:1. The mean age of the patients was 41.2 years, ranging from 20 to 63 years. Idiopathic AVN was the most common risk factor, accounting for 36% of cases, followed by alcohol consumption (32%), steroid use (28%), and trauma (8%). Notably, 34% (n=17) of the patients had a history of COVID-19 prior to AVN symptom onset. Among the 50 cases with 80 hips affected by AVN, the majority (60%, n=30) had bilateral hip involvement, while 22% (n=11) had unilateral right hip involvement and 18% (n=9) had unilateral left hip involvement. The most common chief complaint in our study was hip pain, which was present in 43 (86%) cases. According to the Association Research Circulation Osseous (ARCO) classification of AVN, we observed that stage I AVN was detected in 11 (13.75%) hips, stage II AVN in 19 (23.75%), stage IIIA AVN in 21 (26.25%), stage IIIB AVN in eight (10%), and stage IV AVN in 21 (26.25%).

Conclusion: The ARCO classification system is superior in assessing disease severity. The modified Kerboul's angle is useful for assessing femoral head deformity. The radiologic staging of the disease is of great importance for the identification and risk stratification in pre-collapse stages, prognosis, adequate treatment planning, and post-operative follow-up.

## Introduction

Avascular necrosis (AVN) of the hip, also known as osteonecrosis or ischemic necrosis, is a growing cause of musculoskeletal disability [1,2]. It affects primarily young adults between 20 and 40 years, with an estimated 20,000-30,000 cases per year [3].

AVN is a progressive disorder caused by an insufficient blood supply, leading to cell death, fracture, and collapse of the affected area. Trauma, steroid use, and alcohol are significant contributing factors [4,5].

The blood supply to the femoral head is primarily through the medial circumflex arteries. Blockage of these vessels can lead to AVN [6]. Numerous classification systems, including the Ficat and Arlet classification [6], the Steinberg classification [7], and the Association Research Circulation Osseous (ARCO) [1], have been developed to stage and diagnose AVN.

Imaging is crucial for accurate diagnosis and staging, with modalities including conventional radiography, computed tomography (CT), magnetic resonance imaging (MRI), and nuclear medicine hybrid techniques [6]. Radiologic staging is essential for identifying pre-collapse stages, risk stratification, prognosis, treatment planning, and post-operative follow-up [8]. The present research was therefore conducted to study the role of MRI in the assessment of avascular necrosis of the hip.

## Materials and methods

Aims and objectives

This study aims to investigate the role of magnetic resonance imaging (MRI) in evaluating avascular necrosis (AVN) of the hip, with specific primary objectives to describe MRI-based imaging features and classify AVN using established systems.

The secondary objectives were to assess the risk of femoral head collapse using the modified Kerboul's angle and explore the potential link between COVID-19 infection and AVN development.

Study design and setting

This cross-sectional study was conducted at the Department of Radiodiagnosis, Aarupadai Veedu Medical College and Hospital, Puducherry, over a period of two years from December 2022 to November 2024. A total of 50 cases presenting with suspected unilateral or bilateral hip joint pathology, who met our predefined inclusion criteria, were included in this study.

Inclusion criteria

The study included cases between the ages of 20 and 65 years, of both sexes, referred to the Department of Radiodiagnosis with suspected unilateral or bilateral hip joint pathology, with or without hip pain, for magnetic resonance imaging.

Exclusion criteria

Patients with contraindications to MRI (e.g., metallic implants, cardiac pacemaker, aneurysmal clips, and cochlear implant), other associated hip joint pathologies, previous hip surgeries, claustrophobia, or pregnant women were excluded.

Sample size

Because of the unknown prevalence of osteonecrosis of the femoral head (ONFH) in India, a formal sample size calculation could not be performed. Instead, patients were included in a time-bound manner. The final analysis included 80 hips (50 patients).

Magnetic resonance imaging protocol

All patients in this study underwent MRI scans on a 1.5 Tesla Philips Achieva DS MR system (Philips, New York). The standardized imaging protocol consisted of T1-weighted spin echo (T1W), T2-weighted fast spin echo (T2W), and short-tau inversion recovery (STIR) sequences, acquired in both axial and coronal planes.

Data collection methods

A detailed data collection proforma was filled out to record the necessary details for all participating patients. Consent was obtained from each patient before participation.

Statistical analysis

Statistical analysis was carried out using Statistical Package for the Social Sciences version 28 (IBM Corp., Armonk, NY). All quantitative variables were described using measures of central tendency (mean and median) and measures of dispersion (standard deviation and standard error). Receiver operating characteristic (ROC) analysis was conducted to determine the accuracy of magnetic resonance imaging. A P-value of less than 0.05 was considered statistically significant.

Ethical approval

The study was approved by the Institutional Human Ethical Committee of Aarupadai Veedu Medical College and Hospital, Puducherry, with the approval number AV/IHEC/2023/082.

## Results

Demographic characteristics

The study population had a mean age of 41.2 years, ranging from 20 to 63 years, with a predominantly male demographic (62%, n=31) and a male-to-female ratio of 1.63:1.

Among the 50 cases studied, a total of 80 hips were affected by avascular necrosis (AVN) of the femoral head, with 60% (n=30) of the cases presenting with bilateral AVN, 22% (n=11) with unilateral right hip AVN, and 18% (n=9) with unilateral left hip AVN. The most common clinical manifestations in our study were pain (86%, n=43), followed by limp (60%, n=30), and reduced hip range of motion (34%, n=17) (Table 1).

**Table 1:** Demographic characteristics of the cases. Values are presented as percentages (frequencies). The chi-square test was used for analysis. P-values of <0.001 are considered statistically significant.

Variables	Percentage (%) and number of cases (N=50)		Chi-square value (P-value)
Age distribution (years)			25.4 (<0.001)
20-30		14% (n=7)	
31-40		40% (n=20)	
41-50		32% (n=16)	
51-60		10% (n=5)	
>60		4% (n=2)	
Gender			2.88 (0.0896)
Male		62% (n=31)	
Female		38% (n=19)	
Side affected			16.12 (<0.001)
Bilateral		60% (n=30)	
Right		22% (n=11)	
Left		18% (n=9)	
Clinical symptoms			
Pain		86% (n=43)	
Limp		60% (n=30)	
Range of motion reduced		34% (n=17)	

Associated risk factors and comorbidities

The identified risk factors for avascular necrosis (AVN) in our study included idiopathic factors (36%, n=18), history of alcohol intake (32%, n=16), steroid intake (28%, n=14), smoking (24%, n=12), trauma (8%, n=4), and sickle cell disease/coagulopathy (6%, n=3). Additionally, associated comorbidities were present, including diabetes mellitus (30%, n=15), hypertension (18%, n=9), and high cholesterol levels (20%, n=10). A history of COVID-19 infection was reported in 34% (n=17) of the cases, prior to the onset of AVN symptoms (Table 2).

**Table 2:** Associated risk factors for AVN of the hip and associated comorbidities. AVN: avascular necrosis

Risk factors	Percentage (%) and number of cases (N=50)
Idiopathic	36% (n=18)
Alcohol	32% (n=16)
Steroid	28% (n=14)
Smoking	24% (n=12)
Trauma	8% (n=4)
Sickle cell disease/coagulopathy	6% (n=3)
Associated comorbidities	
Diabetes mellitus	30% (n=15)
Hypertension	18% (n=9)
High cholesterol levels	20% (n=10)

Magnetic resonance imaging findings

**Figure 1:**
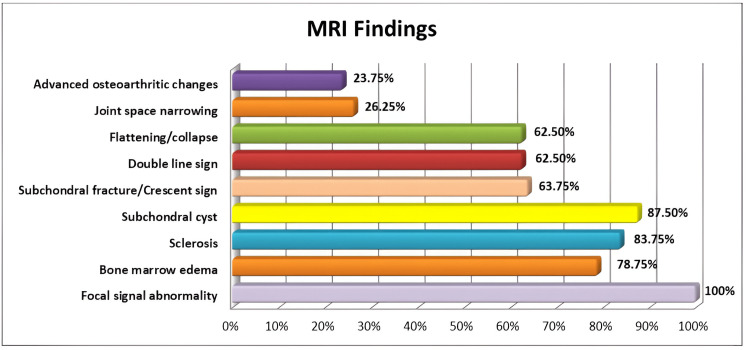
Bar chart showing the distribution of significant findings on MRI of AVN of the hip. Total number of hips involved: 80 MRI: magnetic resonance imaging, AVN: avascular necrosis

**Figure 2:**
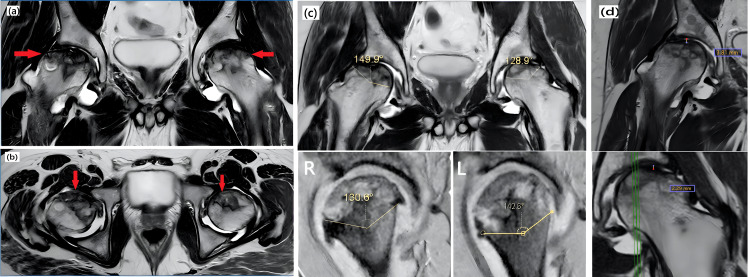
(a) T2W coronal image shows a geographic area of mixed signal intensity with a double line sign bilaterally, predominantly in the antero-latero-superior quadrant extending into the subcapital metaphysis (arrows). Loss of contour of the femoral head with flattening and collapse is seen on the right side. Mild femoral head flattening is seen on the left side. However, the spherical contour is maintained. (b) T2W axial image shows a geographic area of mixed signal intensity with a double line sign bilaterally, predominantly in the antero-latero-superior quadrant, with subchondral cysts (arrows). (c) Modified Kerboul's angle right: -280.5° (149.9°+130.6°); modified Kerboul's angle left: -271.5° (128.9°+142.6°). Findings suggestive of bilateral AVN of the hip joint consistent with ARCO stage IV on the right side and ARCO stage IIIA on the left side. (d) Right hip showing articular collapse of more than 2 mm (3.81 mm) and left hip articular collapse of 2.29 mm. T2W: T2-weighted, STIR: short TI inversion recovery, °: degree, AVN: avascular necrosis, ARCO: Association Research Circulation Osseous

**Figure 3:**
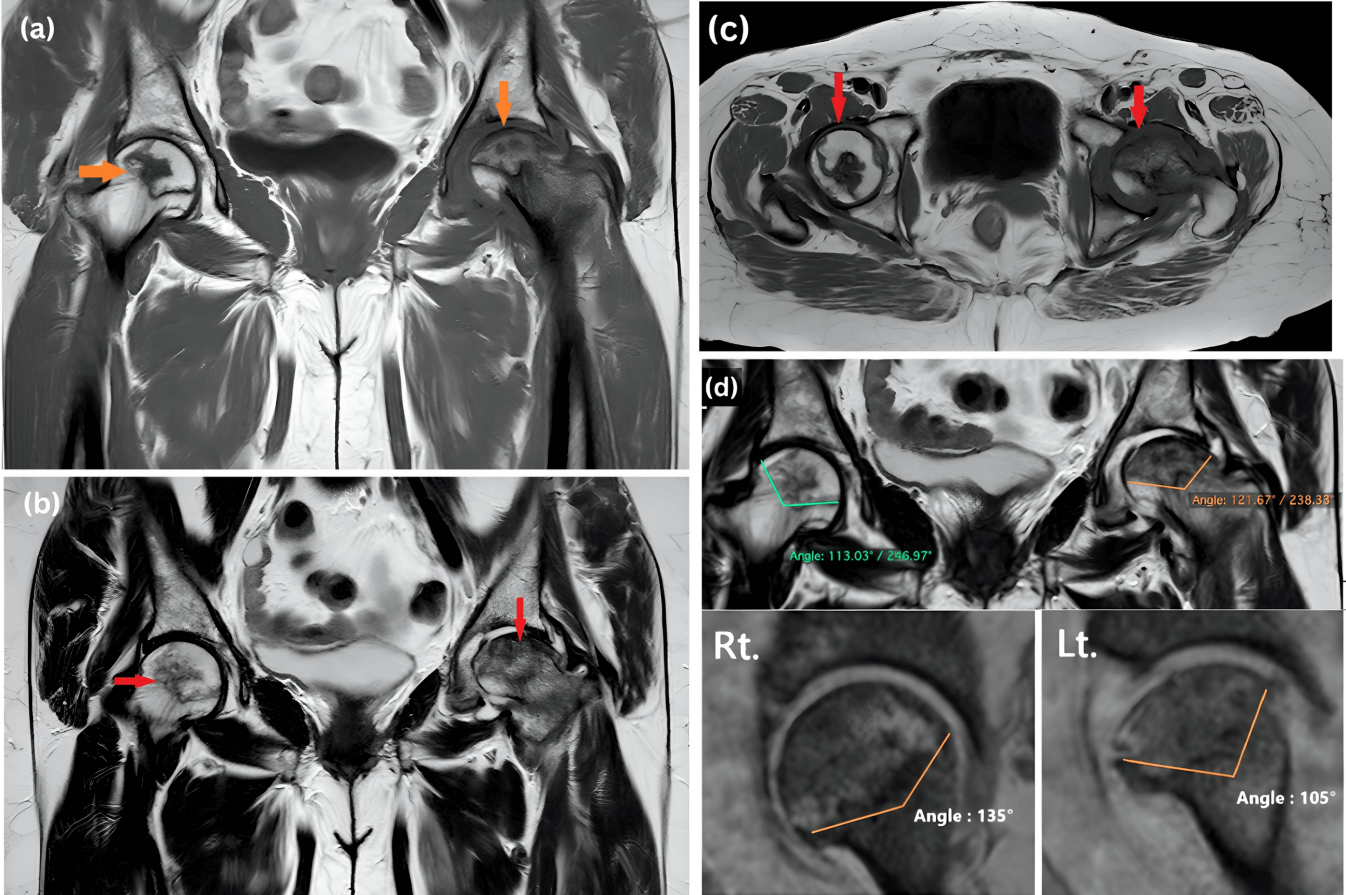
(a) T1W coronal image shows a focal geographic area with a double line sign and subchondral cyst noted on the left side. Significant joint space narrowing is noted. Minimal cortical irregularities are noted in the right femoral head with mild narrowing of the joint space (arrows). (b) T2W coronal image shows bone marrow edema on the left side with mild joint effusion (arrows). (c) T1W axial image shows a geographic area of mixed signal intensity bilaterally, predominantly in the antero-latero-superior quadrant. However, no femoral head collapse was noted (arrows). (d) Modified Kerboul's angle right: -248°(113°+135°); modified Kerboul's angle left: -232° (127°+105°). Findings suggestive of bilateral AVN of the hip joint consistent with ARCO stage II on the right side and ARCO stage IIIA on the left side. T1W: T1-weighted, T2W: T2-weighted, °: degree, ARCO: Association Research Circulation Osseous classification

The most common MRI findings (Figure 1) in our study were focal subchondral abnormality (100%, n=80), subchondral cysts (87.5%, n=70), sclerosis (83.75%, n=67), diffuse marrow edema (78.75%, n=63), double line sign (62.5%, n=50), and flattening/articular collapse of femoral heads (62.5%, n=50) (Figure 2). Additional findings included joint space narrowing (26.25%, n=21) and advanced osteoarthritic changes (23.75%, n=19). Notably, 26.25% (n=21) of the cases had less than 2 mm of contour flattening/articular collapse, while 36.25% (n=29) had more than 2 mm (Figure 3) (Table 3).

**Table 3:** Significant MRI findings in cases of AVN of the hip. Values are presented as percentages (frequencies). The chi-square test was used for analysis. P-values of <0.001 are considered statistically significant. MRI: magnetic resonance imaging, AVN: avascular necrosis

Variables	Percentage (%) and number of cases (N=50)	Chi-square value (P-value)
Contour flattening/articular collapse (n=80)		2.19 (0.335)
Less than 2 mm	26.25% (n=21)	
More than 2 mm	36.25% (n=29)	
Modified Kerboul's angle		
Grade 1 (<200°)	20% (n=16)	0.8 (0.371)
Grade 2 (200°-249°)	37.50% (n=30)	5 (0.025)
Grade 3 (250°-299°)	31.25% (n=25)	1.25 (0.264)
Grade 4 ( ≥300°)	11.25% (n=9)	6.05 (0.014)
Quadrant/location of lesion in femoral head		
Anterosuperior	42.50% (n=34)	
Anteroposterior	32.50% (n=26)	
Anterolateral	5% (n=4)	
Complete	20% (n=16)	

Ficat and Arlet classification

According to the Ficat and Arlet classification [6] of AVN, we observed that stage I AVN (11 femoral heads, 13.75%) revealed diffuse marrow edema in the femoral head and stage II AVN (19 femoral heads, 23.75%) revealed focal geographical area of signal alteration in the subchondral region of the femoral head with double line sign. Stage III AVN (29 femoral heads, 36.25%) revealed disruption of the normal contour of the femoral head with eventual cortical collapse. Stage IV AVN (21 femoral heads, 26.25%) revealed subarticular collapse of the femoral head associated with advanced degenerative changes of the hip (Table 4).

**Table 4:** Stage-wise distribution of AVN of the hip based on MRI findings as per the Steinberg classification, ARCO classification, and Ficat and Arlet classification system. ARCO - Ficat and Arlet classification systems: Spearman correlation coefficient, ρ ≈ 0.95, p < 0.001 ARCO - Steinberg staging: Spearman correlation coefficient, ρ ≈ 0.89, p < 0.001 *P-values of <0.001 are considered statistically significant. AVN: avascular necrosis, MRI: magnetic resonance imaging, ARCO: Association Research Circulation Osseous classification

Steinberg staging			ARCO system			Ficat and Arlet staging	
Stages	Number of hips (N=80)	Percentage (%)		Number of hips (N=80)	Percentage (%)	Number of hips (N=80)	Percentage (%)
Stage 0	0	0		-	-	-	-
Stage I	11	13.75%		11	13.75%	11	13.75%
Stage II	19	23.75%		19	23.75%	19	23.75%
Stage III	12	15%	III A	21	26.25%	29	36.25%
			III B	8	10%	-	-
Stage IV	17	21.25%		21	26.25%	21	26.25%
Stage V	2	2.5%		-	-	-	-
Stage VI	19	23.75%		-	-	-	-

ARCO staging

According to the ARCO classification of AVN [1], we observed that stage I AVN was detected in 11 (13.75%) hips, stage II AVN in 19 (23.75%), stage IIIA AVN in 21 (26.25%), stage IIIB AVN in eight (10%), and stage IV AVN in 21 (26.25%) (Table 4).

Steinberg classification

According to the Steinberg classification of AVN [7], we observed that stage I AVN was detected in 11 (13.75%) hips, stage II AVN in 19 (23.75%), stage III AVN in 12 (15%), stage IV AVN in 17 (21.25%), stage V AVN in two (2.5%), and stage VI AVN in 19 (23.75%) (Table 4).

## Discussion

Our study of 50 patients, comprising 62% (n=31) male patients and 38% (n=19) female patients, revealed a slightly higher incidence of avascular necrosis (AVN) in male patients. This finding is consistent with previous studies by Vardhan et al. [9] and Zhao et al. [10], which also reported a higher incidence of AVN in male patients compared to female patients. The mean age of the patients in our study was 41.2 years (range: 20-63 years), which is comparable to previous studies by Reddy et al. [11], with a mean age of 37.63 years, and Choudhary et al. [12], with a mean age of 38.35 years.

The risk factors for avascular necrosis (AVN) were analyzed, revealing that 32% (n=16) of the cases had a history of alcohol intake in our study, comparable to previous studies by Morge [13], which had a history of alcohol consumption in 45% (n=18) of cases, and Saleem et al. [14], which had a history of alcohol consumption in 56% (n=28) of cases. Similarly, it was present in 46% (n=46) of cases in the study by Choudhary et al. [12]. The exact mechanism of how alcohol leads to AVN is not known; however, according to the findings of George and Lane, the occurrence of bone necrosis due to alcoholism is primarily caused by fat embolism related to existing hyperlipidemia [15]. Therefore, alcohol consumption should be viewed as a major risk factor for the development of avascular necrosis (AVN). In our study, 28% (n=14) of the cases had a history of steroid intake, which is higher compared to similar previous studies by Saleem et al. [14], who reported 14% (n=7), and Morge [13], who reported 10% (n=4). Notably, George and Lane identified fat hypertrophy, intravascular coagulation, and fat embolism as critical risk factors for steroid-induced bone necrosis [15]. Idiopathic factors were identified in 36% (n=18) of the cases in this study, aligning with similar previous studies by Vaghamashi et al. [16] and Kamal et al. [17]. Trauma accounted for 8% (n=4) of the cases in this study, lower than the rates reported in the studies by Choudhary et al. [12], Saleem et al. [14], and Morge [13].

Our study revealed that 34% (n=17) of the cases had a history of COVID-19 prior to the onset of avascular necrosis (AVN) symptoms. Sulewski et al. identified a distinct correlation between COVID-19 and joint changes, with no other risk factors contributing to the development of avascular necrosis (AVN). Notably, the study group was devoid of any pre-existing degenerative or autoimmune diseases. The authors observed a positive response to steroid treatment, characterized by a reduction in joint pain [18]. The study by Namiranian et al. emphasized the importance of vigilant evaluation for AVN in patients recovering from COVID-19 who present with joint pain, highlighting the need for prompt assessment and intervention [19]. High doses of steroids are necessary to save lives due to the severity and danger of the COVID-19 infection, as well as the cytokine storm it generates. According to Panin et al., it is premature to definitively conclude that osteonecrosis is a direct consequence of COVID-19. Instead, the development of the disease is likely influenced by a complex interplay of multiple factors, suggesting a synergistic effect [20]. A systematic review conducted by Migliorini et al. examined the incidence of avascular necrosis (AVN) following COVID-19 infection. The review included nine studies, comprising a total of 245 patients, who developed AVN after contracting COVID-19. The key findings suggested a potential link between the use of short-term, high-dose corticosteroid (CCS) therapy in COVID-19 patients and an increased risk of developing AVN. Notably, the mean time for the development of symptomatic AVN was approximately 80 days. However, due to the high risk of bias in the included studies, the overall quality of the evidence was deemed low, precluding definitive conclusions [21].

Our study found that 60% (n=30) of the AVN cases had bilateral hip involvement. This high prevalence of bilateral hip involvement is consistent with previous studies, such as those by Saleem et al. [14], who reported a bilateral involvement rate of 62% (n=31), and Khaladkar et al. [22], who reported that 61% (n=22) of cases in their study had bilateral involvement and 39% (n=14) unilateral involvement, although it contrasts with the study by Choudhary et al. [12], who reported a higher incidence of unilateral AVN at 68% (n=68). The high prevalence of bilateral involvement in AVN has significant implications for clinical practice. It emphasizes the need for thorough evaluation and management of both hips in patients diagnosed with AVN, rather than focusing solely on the symptomatic hip.

Our study found focal signal abnormality in all 80 hips affected by avascular necrosis (AVN). This finding is consistent with previous studies, including those by Khaladkar et al. [22], Vaghamashi et al. [16], and Morge [13], who also reported a 100% prevalence of focal signal abnormality. Bone marrow edema was observed in 78.75% (n=63/80) of hips in our study. In comparison, previous studies reported the following prevalence rates: Rekha et al., 77% (n=60/65) [23]; Khaladkar et al., 51.70% (n=30/58) [22]; and Vaghamashi et al., 52.20% (n=12/23) [16]. Sclerosis was present in 83.75% (n=67/80) of hips in our study, whereas Rekha et al. reported a lower prevalence of 49.20% (n=32/65) [23]. Subchondral cysts were found in 87.5% (n=70/80) of hips in our study, in contrast with previous studies by Rekha et al. [23], who reported that 43.10% (n=28/65) of cases had subchondral cysts, and another similar study by Vaghamashi et al. [16], who reported 8.70% (n=2/23). Subchondral fractures/crescent signs occurred in 63.75% (n=51/80) of hips in our study. In their study, Rekha et al. reported crescent signs in 50.80% (n=33/65) of cases [23], and Choudhary et al. had 18.2% (n=24/132) of cases with crescent signs [12]. The double line sign was observed in 62.50% (n=50/80) of hips in our study. This finding is consistent with previous studies, which reported prevalence rates ranging from 43.50% to 69.30%. Specifically, Rekha et al. [23] found the double line sign in 69.30% (n=45/65) of cases, Khaladkar et al. [22] reported 56.80% (n=33/58), and Vaghamashi et al. [16] observed 43.50% (n=10/23). In our study, femoral head flattening or collapse was observed in 62.50% (n=50/80) of hips. The prevalence of this feature varied in previous studies, with reported rates of 47.70% (n=31/65) by Rekha et al. [23], 68.90% (n=40/58) by Khaladkar et al. [22], and 56.50% (n=13/23) by Vaghamashi et al. [16]. The prevalence of joint space narrowing in our study was 26.25% (n=21/80). This finding is notable when compared to previous studies. Rekha et al. [23] found joint space narrowing in 30.80% (n=20/65) of cases, Khaladkar et al. [22] reported 37.90% (n=22/58), and Vaghamashi et al. [16] observed 47.80% (n=11/23). Advanced osteoarthritic changes affected 23.75% (n=19/80) of hips in our study. Previous studies by Rekha et al. [23] reported 23.10% (n=15/65), and Vaghamashi et al. [16] reported 26% (n=6/23) of hips with advanced osteoarthritic changes (Table 5).

**Table 5:** MRI findings associated with cases of AVN of the hip in different studies. MRI: magnetic resonance imaging, AVN: avascular necrosis

MRI findings	This study (N=80)	Rekha et al. (2019) [23] (N=65)	Khaladkar et al. (2015) [22] (N=58)	Vaghamashi et al. (2017) [16] (N=23)	Morge (2022) [13] (N=40)	Choudhary et al. (2019) [12] (N=132)
Focal signal abnormality	100% (n=80)	-	100% (n=58)	100% (n=23)	100% (n=40)	69.7% (n=92)
Bone marrow edema	78.75% (n=63)	77% (n=60)	51.70% (n=30)	52.20% (n=12)	55% (n=22)	54.50% (n=72)
Sclerosis	83.75% (n=67)	49.20% (n=32)	-	-	-	-
Subchondral cyst	87.5% (n=70)	43.10% (n=28)	-	8.70% (n=2)	12.50% (n=5)	31.8% (n=42)
Subchondral fracture/crescent sign	63.75% (n=51)	50.80% (n=33)	-	-	-	18.2% (n=24)
Double line sign	62.50% (n=50)	69.30% (n=45)	56.80% (n=33)	43.50% (n=10)	52.50% (n=21)	41.60% (n=55)
Flattening/collapse	62.50% (n=50)	47.70% (n=31)	68.90% (n=40)	56.50% (n=13)	60% (n=24)	-
Joint space narrowing	26.25% (n=21)	30.80% (n=20)	37.90% (n=22)	47.80% (n=11)	45% (n=18)	-
Advanced osteoarthritic changes	23.75% (n=19)	23.10% (n=15)	-	26% (n=6)	17.50% (n=7)	11.30% (n=15)

Our study's findings on the modified Kerboul's angle, a critical measure of femoral head deformity in avascular necrosis (AVN) of the hip, revealed a mean angle of 246.35°, indicating a moderate degree of deformity. Notably, our study's distribution of deformity grades closely mirrored the findings of Boontanapibul et al. [24], which reported a mean modified Kerboul's angle of 239°. Both studies showed a similar trend, with a higher proportion of patients having grade 2 and grade 3 deformity. Our study reported 37.50% (n=30/80) of patients with grade 2 deformity, comparable to the finding of Boontanapibul et al. of 32% (n=16/50), and 31.25% (n=25/80) with grade 3 deformity, consistent with their finding of 28% (n=14/50) [24]. This similarity in findings highlights the importance of the modified Kerboul's angle as a reliable and reproducible measure of femoral head deformity in AVN. The importance of the modified Kerboul's angle lies in its ability to quantify the extent of femoral head deformity, which is a key determinant of patient prognosis and treatment response. By providing a standardized and objective measure of deformity, the modified Kerboul's angle facilitates communication among clinicians, researchers, and patients, ultimately leading to improved patient care and outcomes.

In our study, which included 80 hips affected with AVN, we used the ARCO classification to categorize AVN of the hip into five stages, revealing the following distribution: stage I, 13.75% (n=11); stage II, 23.75% (n=19); stage IIIA (early), 26.25% (n=21); stage IIIB (late), 10% (n=8); and stage IV, 26.25% (n=21). This is consistent with the progression of AVN, where most patients tend to present with intermediate to advanced stages of the disease. When comparing our results with those of Kalekar et al. [25], which involved 45 hips affected with AVN, we observe both similarities and differences. The study by Kalekar et al. [25] also showed a relatively high proportion of stage III cases, with 26.6% (n=12) in stage IIIA (early) and 24.4% (n=11) in stage IIIB (late), and a notable 15.6% (n=7) in stage IV. This pattern aligns with our findings, suggesting that both early and late stage III cases are common in AVN, and the disease progresses rapidly once it reaches these stages. However, there is a marked difference in the stage I distribution between the two studies. In their study, stage I was observed in only 4.4% (n=2) of the patients, which is lower compared to our study's 13.75%, indicating that our cohort might have had a slightly higher representation of early-stage disease. Overall, while the patterns in disease progression (with a higher incidence of stage III and stage IV) are broadly similar between the two studies, the differences in stage I percentages highlight some variability that could be due to these external factors.

The sub-classification of ARCO stage III into IIIA (early) femoral head articular depression ≤ 2 mm and IIIB (late) femoral head articular depression > 2 mm provides a distinct advantage over other classification systems for avascular necrosis (AVN) of the hip. This refined staging enhances the ARCO classification's sensitivity and specificity, particularly in identifying subtle changes in femoral head involvement. The division into stage IIIA and stage IIIB is significant because it allows clinicians to more accurately assess the severity of subchondral collapse, which is a critical factor in determining the prognosis and guiding treatment decisions.

In their study, Zhao et al. [10] and Vezirhüyük et al. [26] emphasized the importance of early-stage AVN classified as ARCO I-II-IIIA (early) in determining the treatment approach and prognosis for patients. According to the study, early-stage AVN typically shows a favorable response to conservative treatment and joint-preserving procedures such as core decompression or vascularized bone grafting, with reported success rates ranging from 70% to 80%. This is because, in the early stages, the femoral head retains a significant amount of structural integrity, and interventions aimed at improving blood flow to the affected area can effectively halt disease progression and delay the need for more invasive procedures. However, in late-stage AVN classified as ARCO IIIB (late) and stage IV, the femoral head experiences significant collapse and structural deformity, making joint-preserving procedures less effective. In these cases, patients often require total hip arthroplasty (THA), a joint replacement surgery, to alleviate pain and restore hip function. The shift from joint-preserving procedures to THA as the disease progresses highlights the critical role of accurate staging in determining treatment options and predicting long-term outcomes. This sub-classification prevents underestimation of early-stage disease and helps identify those at risk for progression, which is not clearly addressed in other systems such as the Ficat and Arlet classification.

Choudhary et al., in their study, compared the ARCO and Ficat and Arlet systems in the MRI-based staging of AVN and concluded that the ARCO classification's subdivision of stage III into A and B offered better predictive value for disease progression and more precise treatment planning. The authors noted that this distinction is especially useful for staging when evaluating the degree of femoral head collapse, a critical parameter that influences the treatment pathway, particularly in deciding whether to pursue conservative management or surgical interventions such as total hip replacement (THR).

Studies have shown that accurate staging directly impacts treatment selection and patient prognosis. However, the variability in classification reliability we observed may lead to inappropriate treatment selection. For instance, misclassifying an ARCO stage IIIA (early) lesion as stage IIIB (late) could result in premature arthroplasty when joint preservation might still be possible. Similarly, underestimating the stage could lead to failed conservative treatment attempts when more aggressive intervention was actually needed. Therefore, improving classification reliability is not merely an academic exercise but has direct implications for patient care and outcomes. The choice of classification system becomes particularly critical when evaluating avascular necrosis risk in surgical treatment.

Recent studies have further reinforced the reliability and clinical utility of the Association Research Circulation Osseous (ARCO) classification system due to its distinct advantages discussed above, and it has gained widespread acceptance in recent years, particularly as MRI-based staging of avascular necrosis (AVN) of the hip. However, despite these advantages, many centers in India continue to rely on older classification systems such as the Ficat and Arlet and Steinberg for staging AVN of the hip. The Ficat and Arlet classification, although historically significant, lacks the level of detail provided by the ARCO system, especially in differentiating between early and pre-collapse stage disease. Similarly, the Steinberg classification does not include MRI findings as comprehensively as ARCO, which has been shown to provide better prognostic value, particularly in identifying subtle changes in femoral head involvement. Given the growing body of evidence supporting the ARCO classification's superiority in predicting disease progression and treatment outcomes, it is essential for healthcare providers in India to consider updating their clinical staging protocols to incorporate this more advanced system. The widespread adoption of ARCO could significantly improve the accuracy of diagnoses, ensure more timely interventions, and ultimately enhance patient care across the country. Moving forward, it is crucial to integrate modern staging systems such as ARCO in both academic curricula and clinical practice guidelines to standardize the approach for AVN management.

Our study has two limitations: a small sample size and limited data. Further studies involving a larger sample size and more data would be helpful in drawing broader conclusions.

## Conclusions

Avascular necrosis (AVN) of the hip is a progressive condition that significantly impacts joint function, often leading to hip joint collapse if not properly managed. This study emphasizes the critical role of MRI in the diagnosis and staging of AVN. One of the key findings of our study is the importance of MRI-based classification systems, particularly the ARCO classification, in assessing the severity of the condition. In conclusion, the use of MRI in the staging of AVN of the hip is indispensable for accurate diagnosis, prognosis, and treatment planning. The ARCO classification stands out for its ability to accurately assess disease progression, differentiate between early and late stages, and guide treatment decisions. As the field of AVN management evolves, embracing modern, MRI-based staging systems such as the ARCO classification will enhance the overall quality of care, ensuring that patients receive timely, effective interventions tailored to the severity of their condition.
